# Improved the expression level of active transglutaminase by directional increasing copy of *mtg* gene in *Pichia pastoris*

**DOI:** 10.1186/s12896-019-0542-6

**Published:** 2019-07-30

**Authors:** Xiaoping Song, Changsheng Shao, Yugang Guo, Yajie Wang, Jingjing Cai

**Affiliations:** 1 Department of Pharmacy, Anhui Medical College, Hefei, 230061 China; 2 Institute of advanced technology, University of Science and Technology of China, Hefei, 230031 China; 3 Anhui Engineering Research Center of Recombinant Protein Pharmaceutical Biotechnology, Hefei, 230022 China

**Keywords:** Transglutaminase, *Pichia pastoris*, rDNA, Pro-peptide, Co-expression

## Abstract

**Background:**

The microbial transglutaminase (MTG) is inactive when only the mature sequence is expressed in *Pichia pastoris.* Although co-expression of MTG and its N-terminal pro-peptide can obtain the active MTG, the enzyme activity was still low. One of the basic steps for strain improvement is to ensure a sufficient level of transcription of the heterologous gene, based on promoter strength and gene copy number. To date, high-copy-number recombinants of *P. pastoris* are achievable only by cloning of gene concatemers, so methods for rapid and reliable multicopy strains are therefore desirable.

**Results:**

The coexpression strains harboring different copies *mtg* were obtained successfully by stepwise increasing Zeocin concentration based on the rDNA sequence of *P. pastoris*. The genome of coexpression strains with the highest enzyme activity was analyzed by real-time fluorescence quantitative PCR, and three copies of *mtg* gene (*mtg-*3c) was calculated according to the standard curve of *gap* and *mtg* genes (*gap* is regarded as the single-copy reference gene). The maximum enzyme activity of *mtg-*3c was up to 1.41 U/mL after being inducted for 72 h in 1 L flask under optimal culture conditions, and two protein bands were observed at the expected molecular weights (40 kDa and 5 kDa) by Western blot. Furthermore, among the strains detected, compared with *mtg*-2c, *mtg*-6c or *mtg*-8c, *mtg*-3c is the highest expression level and enzyme activity, implying that *mtg*-3c is the most suitable for co-expression pro-peptide and MTG.

**Conclusions:**

This study provides an effective strategy for improving the expression level of active MTG by directional increasing of *mtg* copies in *P. pastoris.*

**Electronic supplementary material:**

The online version of this article (10.1186/s12896-019-0542-6) contains supplementary material, which is available to authorized users.

## Background

Transglutaminase (TG, EC2.3.2.13) is an effective protein cross-linker that transfers acyl between glutamine and acyl acceptors to improve the properties of proteins [1–3]. The microbial transglutaminase (MTG) is widely used in the food industry to improve the texture and nutrition [4, 5]. Besides, TG has broad potential applications in biopharmaceuticals [6–9],tissue engineering [3, 7], site-directed protein cross-linking [1, 3, 10], and antibody-drug conjugates [11].

The MTG was produced by *Streptomyces mobaraense* [12], *Streptomyces hygroscopicus* [13], and *Bacillus circulans* [14]. The catalytic active center of MTG consisted of catalytic triad C64-D255-H274 is located at the bottom of its active fracture (Additional file 1: Figure S1). The MTG could be secret to extracellular as a zymogen and the N-terminal peptide was removed by endogenous protease to activate the enzyme [15, 16]. It is hard to express MTG in engineering strains due to the activation mode [17–21]. Some researchers suggested that the MTG is inactive if the full or mature sequence is expressed in *P. pastoris* [17, 21]. To solve the problem, the pro-peptide and mature sequence of MTG could be expressed as two separate elements in *P. pastoris*. The activity of MTG reached 0.25 U/mL and 0.338 U/mL respectively in flasks [22], indicating that the pro-peptide sequence could promote and activate the mature enzyme. However, the activity was too low to meet the industrial requirements. Therefore, the increasing demand on MTG is urgently needed to improve the enzyme activity of MTG.

As we know, the yield of protein is related to the copy number of the target gene [23, 24]. This is a useful method to improve the yield of protein by increasing the copy number of the target gene in yeast [25, 26]. The repeated sequence of rDNA of *P. pastoris* was separated by the non-transcribed intergenic spacer (NTS) [26, 27]. It could be used as the recombination site to increase the copy number of the target gene. In our previous work, the gene of snake venom antiplatelet thrombolytic was integrated into the rDNA repeat sequence. The combinant antiplatelet thrombolytic shared similar physicochemical properties and biological activities with the natural antiplatelet thrombolytic [27]. Therefore, the strategy based on the repeated sequence of rDNA of *P. pastoris* to construct multiple copy of the target gene can be employed in this paper.

In order to improve the expression level and the enzyme activity of the recombinant MTG, genes coding MTG and pro-peptide (*mtg* and *pro*) were optimized to make their mRNA secondary structure more stable according to GC contents and the codon bias of *P. pastoris*. The genes (*pro* and *mtg*) were inserted sequentially into the chromosome of *P. pastoris* at ribosomal DNA repeat sites (rDNA) and the mutant histidinol dehydrogenase site (His 4), respectively, and regulated by the constitutive promoter (*GAP*) and the methanol-induced promoter (*AOX*1), respectively. A high coexpression of pro-peptide and MTG was obtained by high-pressure screening of antibiotic and optimization of fermentation conditions. To our knowledge, this is a new report of improving the expression level of active MTG by directional increase of the copy of *mtg* gene in *P. pastoris.*

## Results

### Construction balanced co-expression strains for pro-peptide and MTG

**Fig. 1:**
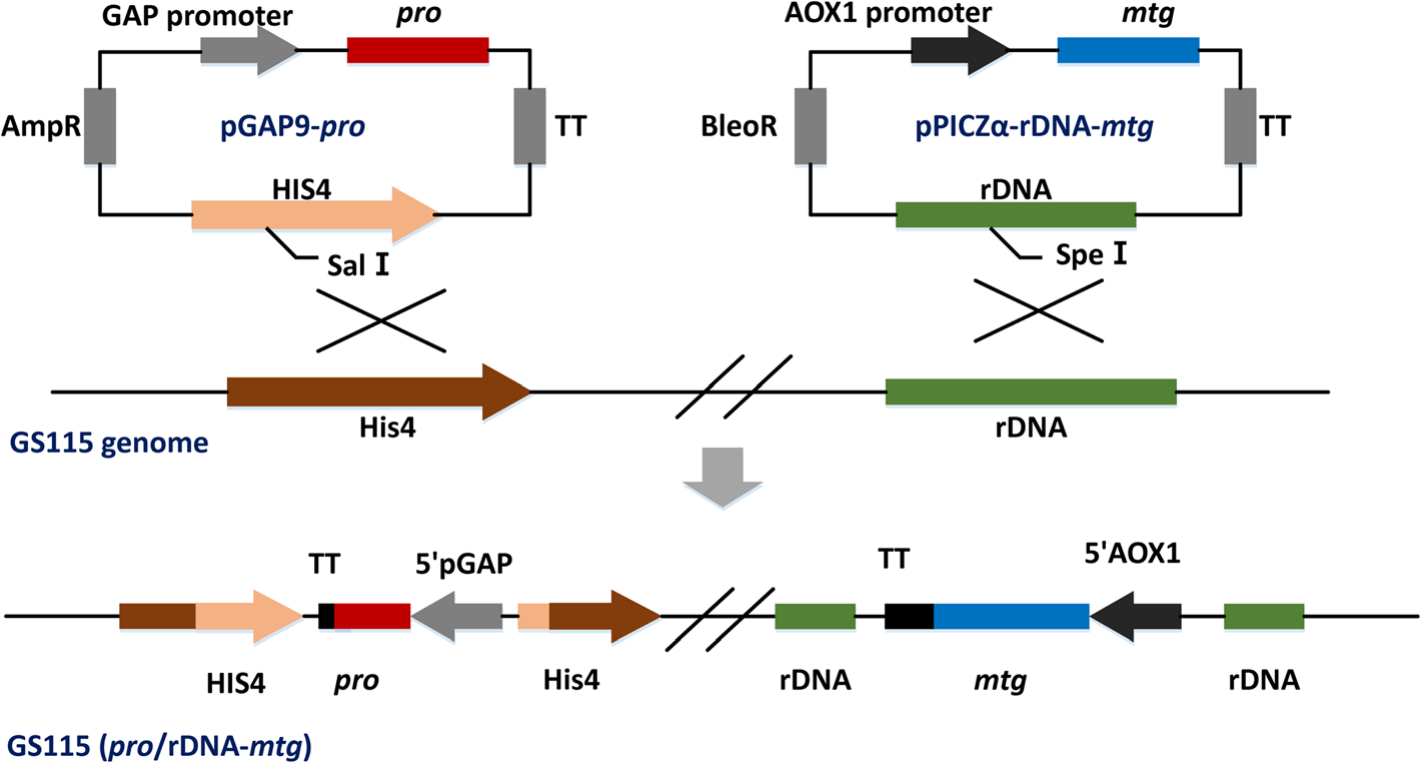
Strategy for the construction of *mtg* and *pro* co-expression strains. This schematic map represents the constructed expression vectors for the *pro* and *mtg,* designated pGAP9/*pro* and pPICZα-rDNA/*mtg*, respectively. The his4 and rDNA non-coding sequences allow the vector to be inserted into the corresponding sites in the genome of strain GS115 through homologous recombination. The pPICZα-rDNA*-mtg* vector was constructed by introducing a non-coding rDNA (indicated as rDNAnc) sequence into the pPICZα vector. The expression of both *pro* and *mtg* were under the control of the GAP and AOX1 promoters, respectively

The genes (*pro* and *mtg*) were inserted into vector pGAP9 and pPICZα to construct the expression vector pGAP9-*pro*and pPICZα-rDNA-*mtg*, respectively (Fig. 1). Both *pro* and *mtg* genes could be integrated into the his4 site and non-coding rDNA sequence site when vectors were transformed into the host strain GS115, respectively. The genes (*pro* and *mtg*) were fused with the *S. cerevisiae* α-mating factor signal sequence and placed under the regulation of GAP and AOX1 promoters. The recombinants of co-expression strain GS115 (*pro*/rDNA*-mtg*) were analyzed by PCR (Additional file 4: Figure S4), of which the results implied that the *pro* and *mtg* genes were integrated into the desired location of *P. pastoris* genome.

**Fig. 2:**
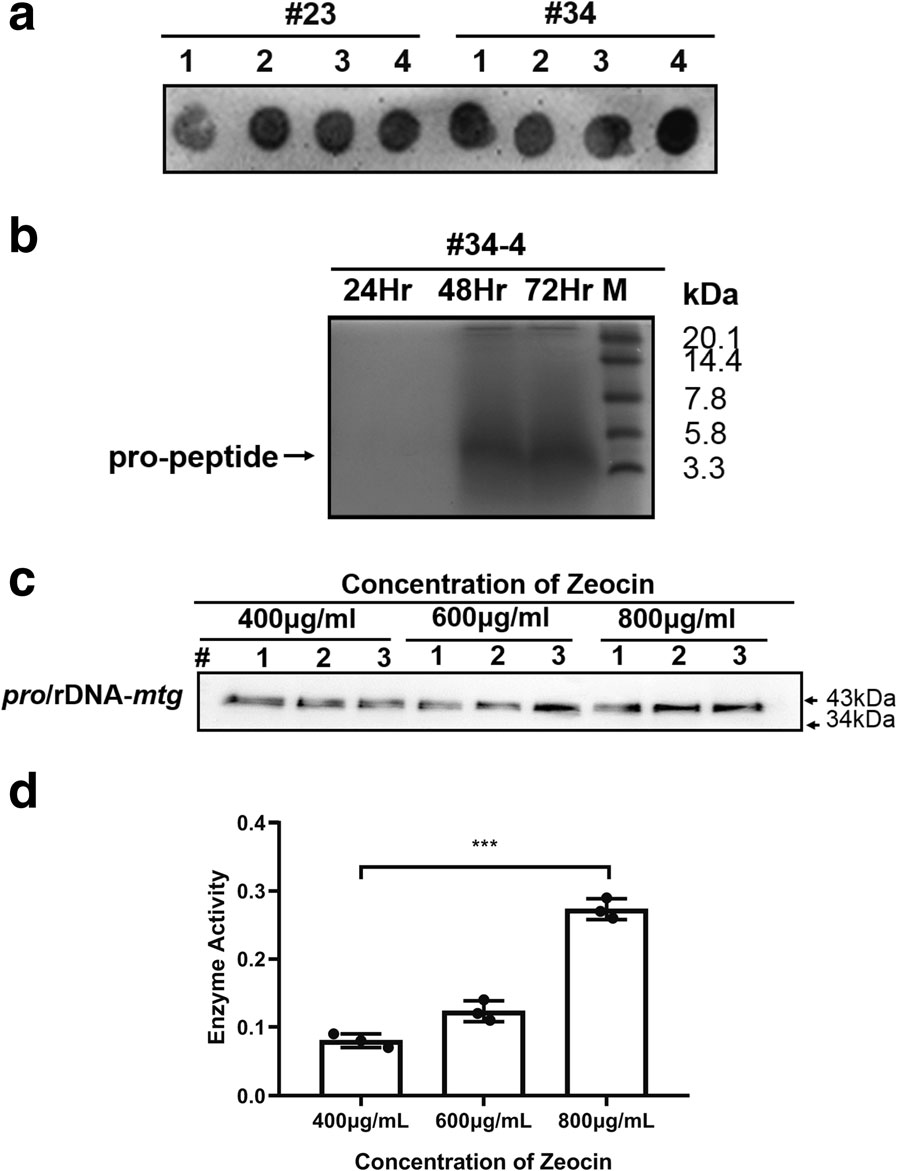
Establishing balanced expression strains for pro-peptide and MTG. **a** The clones of GS115 (*pro*) were screened by Dot blot (anti-His-HRP).1–4 were four repeated of clones. **b** The protein (pro-peptide) of 20 μl culture supernatant was separated by Tricine-SDS-PAGE and stained with Coomassie blue under non-reducing conditions. **c** The co-expression of pro-peptide and MTG from screened colonies with various doses of Zeocin were experimented by Western blot under non-reducing conditions. **d** The enzyme activity of MTG from screened clones with various doses of Zeocin were experimented. Three individual clones were tested for each condition.***Represents a statistically significant difference of *P* < 0.001 compared with other two antibiotic dosage groups

To generate the coexpression strain of pro-peptide and mature MTG, the expression plasmid (pGAP*-pro*) was transformed into *P. pastoris* GS115 and selected by histidine-deficient minimal dextrose (MD) plates, while the clone (# 34–4) with the highest expression of pro-peptide was selected (Fig. 2a and b) and used for the host strain of the co-expressed (*pro*/rDN*A-mtg*). The clones with higher co-expression were selected in MD plate (contain 400 μg/mL, 600 μg/mL, 800 μg/mL Zeocin) and the expression of MTG was measured by Western blot with anti-MTG antibody employed. All selected clones rendered positive results by Western blot and showed an increasing trend with the increase of antibiotic dose (Fig. 2c). The enzyme activity of clone (contain 800 μg/mL Zeocin) reached up to 0.27 U/mL, higher than those of the other two antibiotic dosage groups (0.08 U/mL, 0.12 U/mL) (Fig. 2d).

### Copy number of *mtg* gene in *P. pastoris* genome

**Fig. 3:**
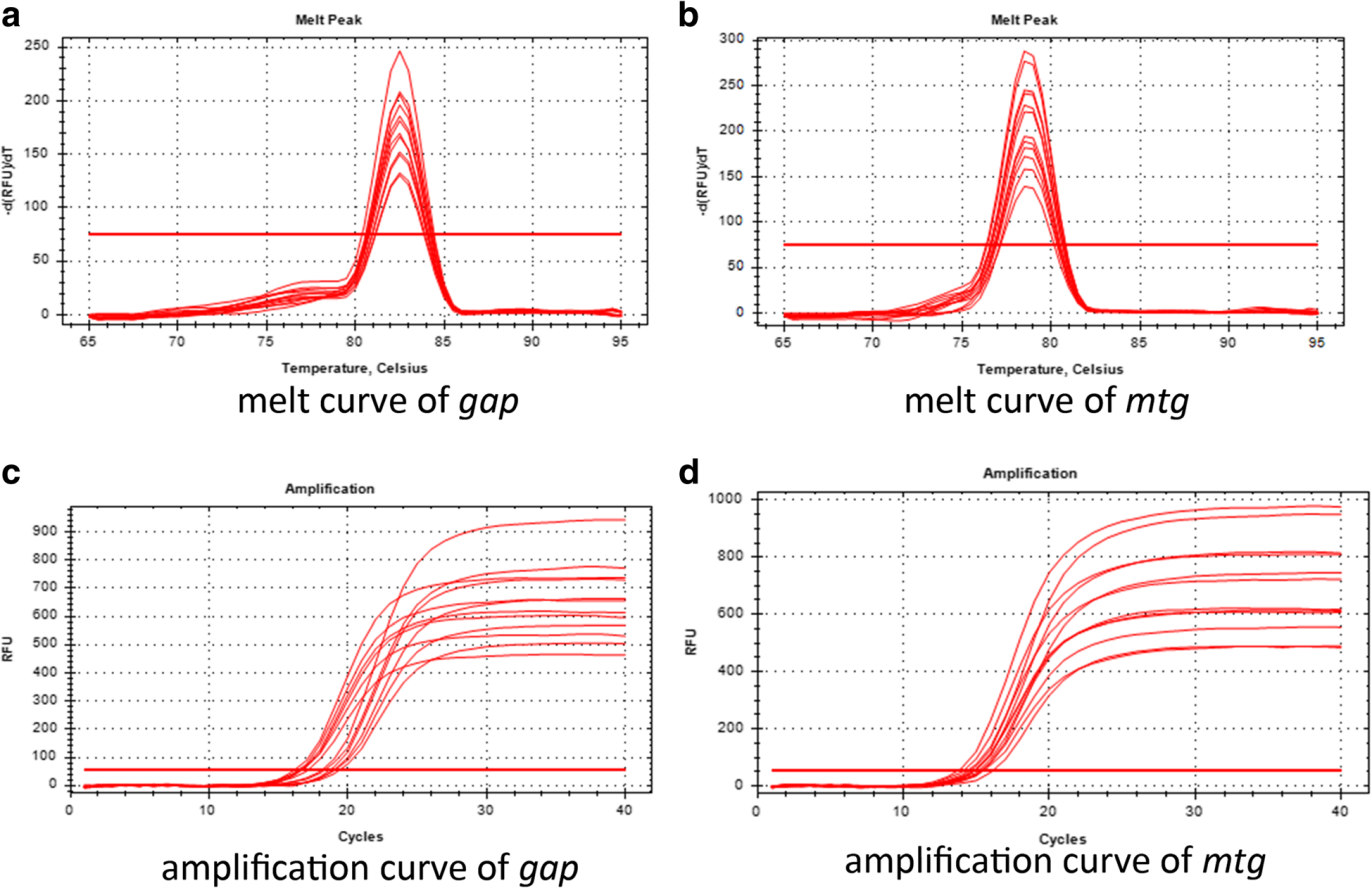
Detection of *mtg* copy number in *P. pastoris* genome by absolute quantitative PCR. **a** and **b** The melt curves of *gap* and *mtg* genes. **c** and **d** The amplification curves of *gap* and *mtg* genes

The results of *mtg* copy number in *P. pastoris* genome are shown in Table 1. The amplification and melt curves of *gap* and *mtg* genes are shown in Fig. 3 while the standard curves are shown in Additional file 5: Figure S5. Both the melt curves of *gap* and *mtg* genes have only one single peak, indicating specificity of the PCR products (Fig. 3a, b). The amplification curves of *gap* and *mtg* genes were shown in Fig. 3c and d. The regression equations of both *gap* and *mtg* genes standard curve were y = − 3.6892x + 35.747 (R = 0.9985), y = − 3.4511x + 33.968 (R = 0.9985), respectively. By the slope of the two standard curves, the response efficiency of *gap* and *mtg* genes were calculated as 86.66 and 94.88%, respectively, indicating similar results of the two ones. The result of *mtg* gene copy number in *P. pastoris* (with the highest activity) is shown in Table 1, demonstrating that *mtg* gene existed in the genome in the form of 3.36 copies and that 3 copies *mtg* gene were directed to the rDNA site in *P. pastoris* and named *mtg*-3c. In addition, the copy numbers of *mtg* gene in the other three detected strains were 2.21, 5.72 and 7.62, respectively (named *mtg*-2c, *mtg*-6c, *mtg*-8c) (Table 1).

**Table 1:** Copy numbers of *gap* and *mtg* gene detected by real-time fluorescent quantitative PCR

Strain	Value Ct		Gene copy(10^n^)		Number of copies of *mtg* gene in *P. Pichia* genome (*mtg* copy number/*gap* copy number)
	*mtg* gene	*gap* gene	*mtg* gene	*gap* gene	
1	14.14 ± 0.27	16.49 ± 0.17	5.58 ± 1.02	1.66 ± 0.19	3.36 ± 0.24
2	14.93 ± 0.28	16.67 ± 0.22	3.29 ± 0.50	1.48 ± 0.18	2.21 ± 0.11
3	14.97 ± 0.30	18.23 ± 0.23	3.20 ± 0.63	5.60 ± 0.71	5.72 ± 0.35
4	15.30 ± 0.41	19.05 ± 0.17	2.56 ± 0.55	3.36 ± 0.31	7.62 ± 1.11

Datas are presented as mean ± SD of triplicate observations. Strain 1: the clone with the highest enzyme activity. Strain 2,3,4:another three positive clones

### Effects of temperature and pH on growth and enzyme activities of co-expressing strains

**Fig. 4:**
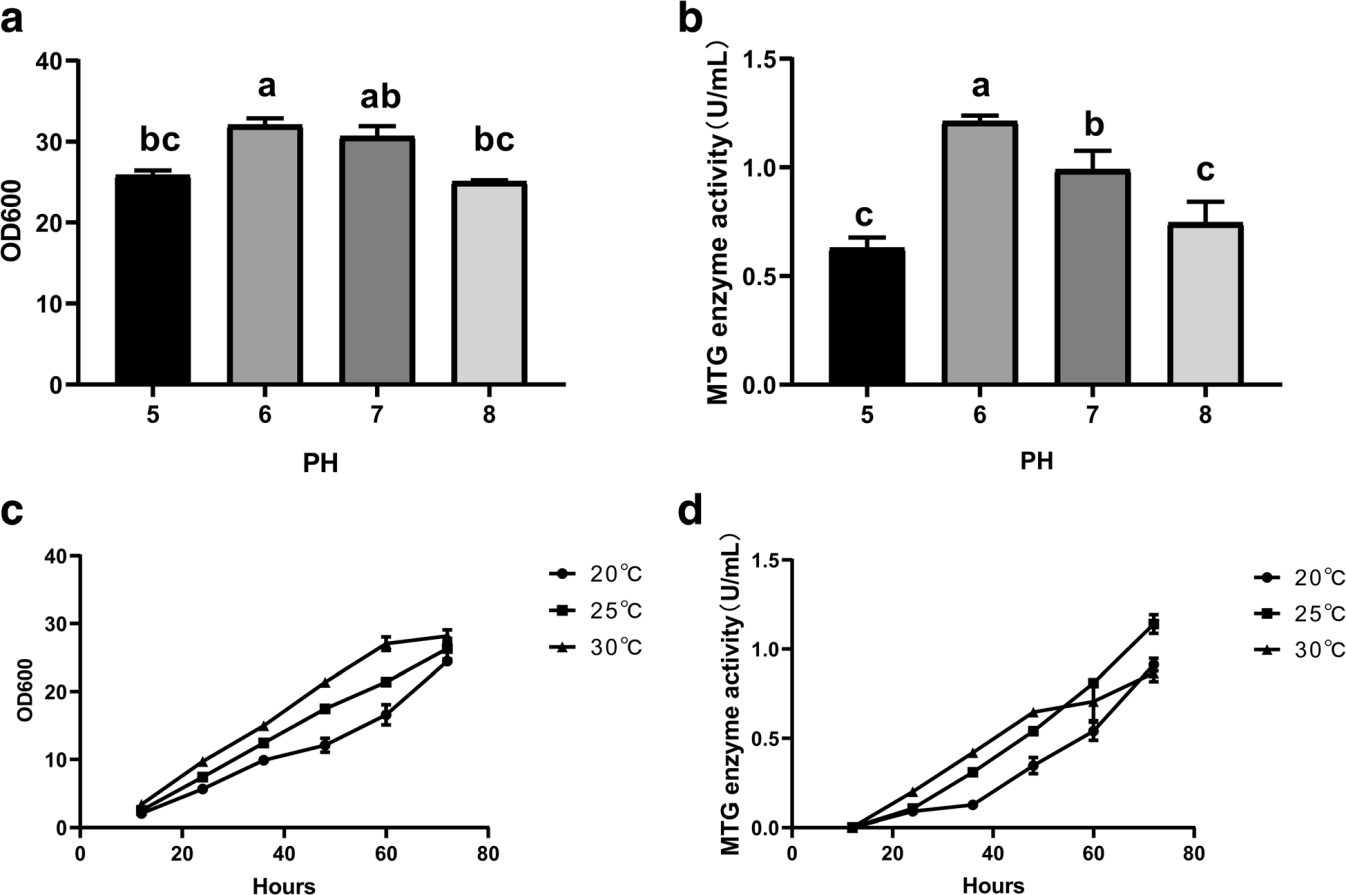
Effects of inducted temperature and pH on growth of GS115 (*pro*/rDNA-*mtg*) and enzyme activities of MTG. **a** and **b** were the effect of different pH on the strain growth and enzyme activity of MTG. **c** and **d** were the effect of different induced temperature on the growth and enzyme activity of MTG. Datas of strain growth and enzyme activity are presented as mean ± SD of triplicate observations. Alphabets (**a**–**c**) in superscript donate significant difference (*P* < 0.05)

The engineering strain *mtg*-3c (*pro*/rDNA*-mtg*) was cultured in 200 mL BMMY medium at different pH, with the result suggesting that the initial pH of the medium exerted minor influence on the cell growth but significant one on the enzyme activity of MTG as illustrated in Fig. 4a, b. The activity of MTG was higher at pH 6 and 7 than that at pH 5 or 8, indicating that the neutral environment was conducive to producing enzymes or the enzyme was more stable in neutral environment. Furthermore, the strain GS115 (*pro*/rDNA*-mtg*) was inducted at various temperatures as shown in Fig. 4c, d. The activity of MTG at 25 or 30 °C was higher than that at 20 °C within 48 h. However, the activity of MTG at 30 °C decreased after 48 h. The highest activity of MTG was 0.91, 1.12, and 0.81 U/mL at 20, 25, and 30 °C, respectively for 72 h, indicating that the optimum inducted temperature was 25 °C. The activity of MTG would increase significantly by controlling the fermentation conditions (the initial pH of BMMY medium and inducted temperatures).

### Co-expression of pro-peptide and MTG in 1 L flask

**Fig. 5:**
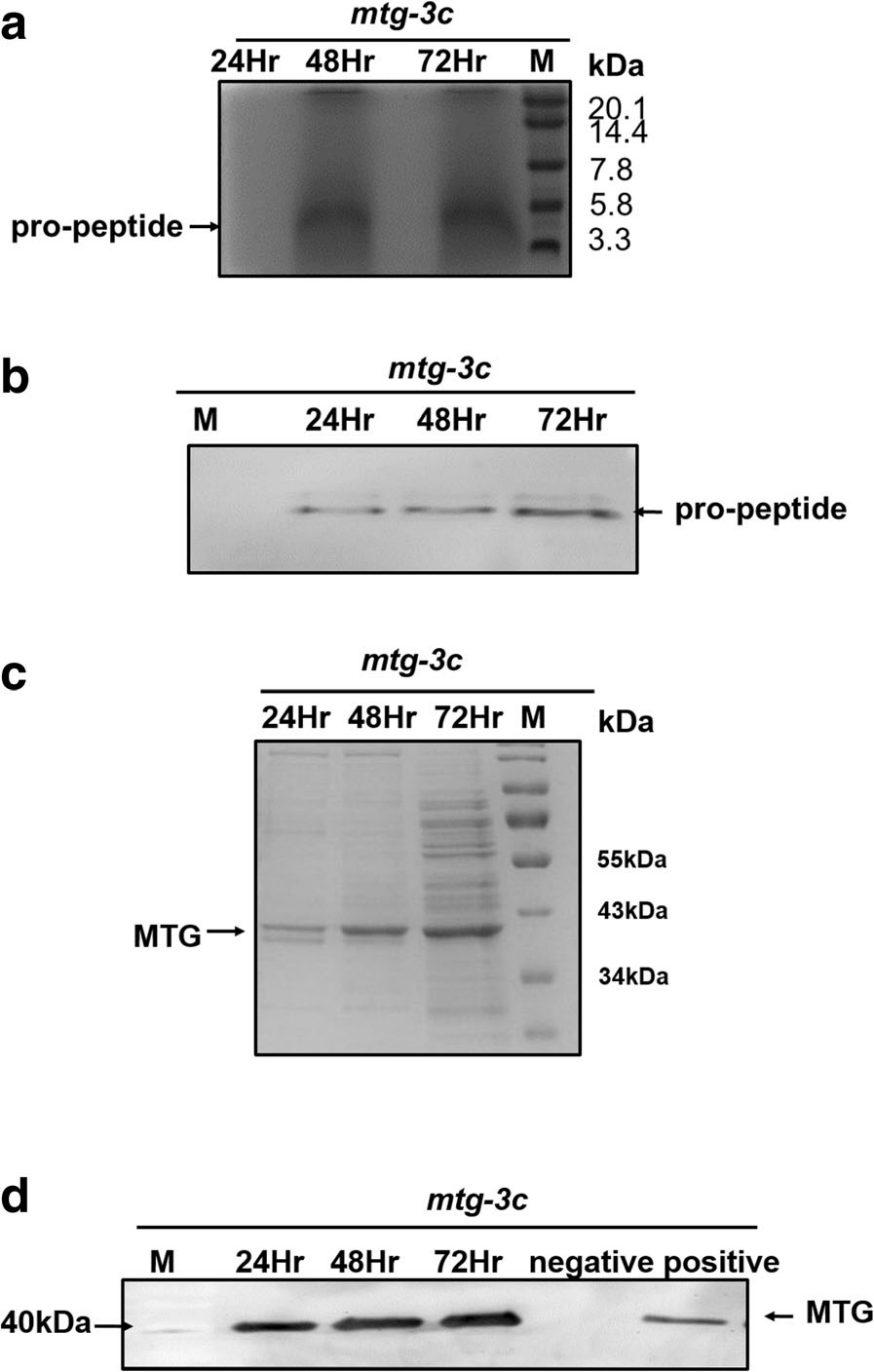
Detection of coexpression pro-peptide and MTG in GS115 (*mtg-*3c). **a** and **b** The pro-peptide were detected by SDS-PAGE and Western blot under non-reducing conditions. The proteins (pro-peptide) of 20 μl culture supernatant were separated by Tricine-SDS-PAGE and subjected to Western analysis using anti-His-HRP. **c** and **d** The protein (MTG) of 20 μl culture supernatant were separated by SDS- PAGE and subjected to Western analysis (anti-MTG).positive control: MTG from *E*.*coli*

The engineering strain *mtg*-3c was inoculated into BMGY medium for 24 h and then transferred into 200 mL BMMY medium. The pro-peptide and MTG in the supernatant have been evaluated by SDS-PAGE electrophoresis and Western blot, respectively (Fig. 5). The experimental results showed that one protein band was observed at the expected molecular weight (40 kDa) (Fig. 5d). In addition, another protein band was observed at the expected molecular weight (5 kDa) by Tricine-SDS-PAGE (Fig. 5b). Because the molecular weights of pro-peptide (5 kDa) and MTG (40 kDa) are significantly different from each other, it is difficult to display them on the same SDS-PAGE gel (Fig. 5a, c). The strategy of combining two homologous recombination sites (His4 and rDNA repeat site) could enable the co-expression of pro-peptide and MTG in *P. pastoris.* The enzyme activity of *mtg*-3c (culture supernatant) was 1.41 U/mL after being inducted for 72 h.

## Discussion

The *P. pastoris* has many outstanding advantages as a host for the expression of foreign proteins [2, 18]. It enjoys the advantages of the strong inducing promoter AOX1, high protein expression, stable genetic traits, simple composition of the medium, etc. Various exogenous genes such as interferon, HBsAg, phytase, albumin, antibody and collagen were expressed in *P. pastoris* successfully [18, 21, 27]. Some researchers have attempted to express recombinant *Streptomyces* MTG in *P. pastoris*; however, the yield of MTG is very low [2, 18, 21]. We also encountered the problem of low yield of recombinant MTG in the previous research. In order to increase the yield of recombinant MTG in *P. pastoris*, we constructed 3 copies *mtg* of GS115 (*pro*/rDNA-*mtg*) with higher expression, and optimized such conditions of protein expression as pH and temperature to increase the yield of MTG.

The copy number of the target gene is an important factor for the yield of the target protein in *P. pastoris* [23]. The expression of the target protein could be effectively boosted by increasing the copy number to 4–9 of the gene [17]. When the copy number was more than nine, the yield of target gene might decrease because the high concentration of target protein had a negative effect on the strain growth and metabolism [25, 28]. In this study, we constructed a high expression strain harboring different copies *mtg* by directionally increasing *mtg* gene copy in *P. pastoris,* and verified the influence of gene dosage on the expression level of MTG. The absolute quantitative PCR analysis was successfully applied to quantify *mtg* copies in the recombination strains. Among the clones being detected, *mtg* genes existed in the *P. pastoris* genome in the form of 2,3,6 or 8 copies, respectively (named *mtg*-2c,*mtg*-3c,*mtg*-6c, *mtg*-8c). Under the same culture conditions (200mLBMMY in shake flask), the effect of different copy *mtg* genes on protein expression and enzyme activity was shown in Additional file 6: Table S1 and Figure S6. The experimental datas showed that the expression levels of *mtg* gene varying with copy numbers were inconsistent, and fell into the range of was *mtg*-3c > *mtg*-2c > *mtg*-6c (Additional file 6: Figure S6). The results revealed that the recombinant strain produced the enzyme faster in case of no more than 3 copies of *mtg* gene, showing the positive effect of gene copy number. However, when gene copy number is more than 3, the enzyme production of the strain dwindled for the excessive protein load, resulting in more heteropolymeric protein and a sharp decrease in enzyme activity than one of the low-copy strain [28, 29]. This fact demonstrated the negative effect of gene copy number, which may be attributed to the burden of the excessive copy number on the cell, resulting in the metabolic imbalance of the strain itself. For this reason, the multiple copies may not result in a high expression level. These results imply that other factors limited the transcription, translation, and secretion of MTG, indicating that the copy number was not necessarily related to the expression level, and that the reasonable copy number was beneficial to improving the protein expression level. These results revealed that 3 copies of *mtg* gene were the most suitable for *pro* and *mtg* co-expression in this study.

The result of culture conditions optimization showed that the initial pH of medium has significant influence on the enzyme activity of MTG. The optimum pH of MTG expression is between 6.0 and 7.0 and the stability of MTG is poor under acid and alkaline conditions, especially under acid conditions (pH < 6.0). The activity of protease produced by *P. pastoris* is suppressed at the pH 6.0–7.0, which could reduce the degradation of the target protein [28]. Furthermore, the temperature would affect the yield of MTG, which is lower at 30 °C than that at 20 or 25 °C, indicating that the rate of cell growth is lower at 20 and 25 °C, but the yield of MTG is higher in this case, attributable to the hypothermia reducing the residence time of the protein in the endoplasmic reticulum, which is conducive to the correct folding of the target protein [30, 31].

Finally, the engineering strain GS115 (*pro*/rDNA-*mtg*) was cultured in 200 mL BMMY medium for 72 h. The maximum enzyme activity of supernatant was 1.41 U/mL, 3.17 times higher than that of a coexpression strain as single-copy expression cassettes (0.338 U/mL) [22]. In addition, two protein bands were observed at the expected molecular weight (40 kDa and 5 kDa) by Western blot, indicating that the co-expression of pro-peptide and MTG can directly secrete active MTG. This is a new report of improving the expression level of active MTG by directional increasing copies of *mtg* gene in *P. pastoris* in the published articles .

The pro-peptide is essential for protein folding, playing the main roles of helping proteins form compact structures and thus reducing the steric hindrance of proteins, guiding proteins to correctly fold and forming active mature enzymes [21]. In this study, the expression level of MTG was mainly measured by enzyme activity. Therefore, the enhanced expression of pro-peptide will facilitate the correct folding of MTG, thus forming much more active MTG. However, the balance of co-expression of pro-peptide and MTG is a problem to be solved in this study. The balance expression of MTG and pro-peptide can be achieved by gradually increasing the concentration of Zeocin antibiotics.

## Conclusions

Based on the non-transcriptional rDNA sequence of *P. pastoris*, the co-expression strain (*pro*/*rDNA-mtg*) with three copies *mtg* genes was constructed successfully. However, the expression in *P. pastoris* is at the milligram per liter level in this paper, which needs to be raised to grams per liter for industrial applications. Successful co-expression of MTG and pro-peptide as well as optimization of their ratios is a challenge, but worth investigating.

## Methods

### Strains and vectors

Strains and vectors were described in Table 2. The E. coli was cultured in Luria-Bertani (LB) medium at 37 °C, whereas the *P. pastoris* was cultured in buffered glycerol-complex medium (BMGY) or buffered methanol-complex medium (BMMY) at 28 °C or 25 °C, respectively.

**Table 2:** Strains and plasmids used in this study

Strains	Genotype	References
*E. coli* TOP10	F-*mcrA*Δ (*mrr-hsdRMS-mcrBC*) φ80 *lacZ*ΔM15 Δ*lacX*74 *recA1 ara*Δ139 Δ (*ara-leu*)7697 *galUgalKrpsL* (*StrR*) *endA1 nupG*. used for vector sub-cloning	Invitrogen
*Pichia pastoris* GS115	*his4*,host strain	Invitrogen
GS115 (*pro*)	The pro-peptide of MTG was integrated into his4 site of *Pichia pastoris* GS115	This study
GS115(*pro*/rDNA-*mtg*)	The mature sequence of MTGwas integrated into non-coding rDNA site ofGS115 (*pro*)	This study
Plasmids	Description	Reference
pPICZα-B	Containing AOX1 promoter for tightly regulated, methanol-induced expression of the gene, Zeocin^R^	Invitrogen
pGAP9	pPIC9 derivative, GAP promoter instead of AOX1 promoter	Lab stock
pGAP9-*pro*	pGAP9 derivative carrying an internal 135 bp fragment of *pro* gene	This study
pPICZα-rDNA-*mtg*	pPICZα-*mtg* derivative carrying an internal 1153 bp fragment of rDNA gene, Zeocin^R^	This study

### Synthesis of *mtg* and *pro* genes

The *mtg* and *pro* genes were selected from the cDNA of *S.mobaraensis* (NO. DQ132977) [19, 21] and synthesized after the codon optimization based on the *P. pastoris* preference (Additional file 2 and Additional file 3: Figures S2 and S3). The primers were designed by Primer premier 5(http://www.premierbiosoft.com/primerdesign/index.html) as shown in Table 3.

**Table 3:** PCR primers used in the present study

Primer name	Primer sequence (5′ → 3′)
P1	ACACTCGAGaaaagaGACTCCGACGACAGGGTCAC
P2	TATGCGGCCGCTCAATGGTGATGGTGATGATGCGGCCAGCCCTGC
P3	ACACTCGAGaaaagaGACAAT GGC GCG GGG GAAG
P4	TATGCGGCCGCTCAATGGTGATGGTGATGATGGGGAGCCCGGAACG
P5	TATGCGGCCGCCAGCTTTCTAGAACAAAAACTCATCTC
P6	CTTAAATATTAGGAAAAACGGTAACCTTATCTCACTTAATCTTCTGTACTCTG
P7	CAGAGTACAGAAGATTAAGAGAGATAGTTAGGTTACCGTTTTTCCTAATATTTAAG
P8	CGCGGATCCCTTCCACCAACAGTCAACCACCAGTC
P9	GGTATTAACGGTTTCGGACGTATTG
P10	GATGTTGACAGGGTCTCTCTCTTGG
P11	TGAAGAAAGAATTGGCTAACGG
P12	AGCTGGTCTGAAAGCATCTGG

Remarks: The underlined sites are those for the digestion of restriction enzymes *Xho*I. The wavy line sites are those for the digestion of restriction enzymes *Not*I. The double corrugated underlined sites are the ones for the digestion of restriction enzymes *Bam*HI. 6xHis-tag label sequence is indicated by the dotted line. The Kex2-endopeptidase recognition site is marked with lowercase letters. The bold underlining represents the sequence of overlapping segments of the AOX1 terminator-rDNA fusion gene

The restriction enzyme site (*Xho*I) and the Kex2 endopeptidase recognition site were added to the 5′-terminus of MTG and pro-peptide (primer P1 and P3). The restriction enzyme site of *Not*Iand the sequence of histidine tag were added to the 3′-terminus (primer P2 and P4).

### The construction of rDNA-mediated *mtg* multi-copy expression vector

The structure of expression pPICZα-rDNA-*mtg* was shown in Fig. 1.The AOX1-terminator (primer P5and P6) and non-coding rDNA fragment (primer P7and P8) were amplified and fused to form the AOX1-rDNA by overlapping PCR as follows: a program consisting of 30 cycles at 95 °C for 40 s, 54 °C for 50 s and 72 °C for 120 s. Then the AOX1-rDNA and the vector pPICZa-*mtg* were digested by *Not*I and *Bam*HI and connected by T4 DNA ligase. The recombinant vector (pPICZα-rDNA-*mtg*) was transformed into *E. coli* TOP10 and sequenced to ensure base sequence was not mutated. The same method was used to construct the control group (pPICZα-*mtg).*

### Construction of the pro-peptide recombinant strain

The plasmid pGAP9 and pro-peptide gene were digested with *Xho*I and *Not*I, ligated by DNA T4 ligase. Then it was transferred into *E. coli* TOP10 competent cells to construct the vector pGAP9-*pro*. It was digested by *Sal*I and electro-transformed into host strain GS115. The recombinants were selected in MD plate without histidine and cultured in BMGY medium for 72 h. The pro-peptide in the supernatant was identified by Dot blot with the antibody anti-His-HRP. Furthermore, the pro-peptide was measured by Tricine-SDS-PAGE. The engineering strain was named GS115(*pro*) and used as the host strain of co-expressed pro-peptide and MTG.

### Construction and selection of the co-expression strain

Recombinant vectors (pPICZα-rDNA-*mtg*) were linearized by *Spe*I, and transformed into the host strain GS115 (pro-peptide). The clones with higher expression were selected by stepwise increasing the Zeocin concentration by 400 μg/mL, 600 μg/mL and 800 μg/mL on MD plates, respectively, so as to strike a better balance of the co-expression of MTG and pro-peptide. The clone with the highest expression was selected in MD plate (contain 800 μg/mL Zeocin) and named GS115 (*pro*/rDNA*-mtg*), respectively. Then they were cultured in BMGY medium for 24 h until the OD600 reached 6.0. The cell was harvested and induced in 5 mL BMMY medium containing 1% methanol for 72 h. The MTG in the supernatant was identified by Western blot (the antibody: Anti-MTG) as described by “Molecular cloning: A laboratory manual”. A clone with the highest enzyme activity was selected.

### Analysis of *mtg copy* number by absolute quantitative PCR

The clones with the highest enzyme activity and other three positive ones were selected for analyzing copy number of *mtg* gene, which in *P. pastoris* genome were determined by real-time fluorescent quantitative PCR (qPCR) [32]. The housekeeping gene *gap* exists as a single copy in the genome of *P. pastoris* [29], so the copy number of *gap* gene can be used to represent the initial copy number of the genome in the template.

The standard curves o*f gap* an*d mtg* genes were prepared with the standard plasmids containin*g gap* an*d mtg* genes, respectively. Both *gap* an*d mtg* genes were amplified by qPCR with the genome of *P.pastoris* transfected *mtg* gene used as template, respectivel*y* (*gap* primer: P9 and P10*, mtg* primer: P11 and P12). Each sample was detected three times, and three parallel reactions were set for each time. The *Ct* values of *gap* and *mtg* genes obtained substituted for the corresponding standard curves, and then the initial template copy number of *gap* and *mtg* genes in DNA samples was calculated. The ratio of initial template copy number of *mtg* and *gap* genes was the copy number of *mtg* gene in *P. pastoris* genome. The reaction system is as follows: a total of 10 μl reaction system, including SYRB Green Realtime PCR Master 5 μl, DNA template 1 μl, each primer 0.5 μl (10 μmol·L^− 1^), H_2_O 3 μl*.* The reaction procedures: 10 mins at 95 °C, followed by 40 cycles of 10 s at 95 °C, 30 s at 60 °C*.*

### Optimization of culture conditions for engineering strain *pro*/rDNA-*mtg*

The strain GS115 (*pro/*rDNA*-mtg*) of higher enzyme activity was cultured in 100 mL BMGY medium for 24 h and controlled at 28 °C. Then the cell was harvested by centrifugation and inoculated into 200 mL BMMY medium at different initial pH (5.0, 6.0, 7.0 and 8.0) for 72 h and maintained at 25 °C. 1 M Na_2_HPO_4_•12H_2_O (pH = 8.85) and 1 M KH_2_PO_4_ (pH = 4.01) were mixed in a certain proportion to form phosphate buffer with buffer range pH 4–9 and used to adjust the initial pH of BMMY medium. The cell density (OD600) and the activity of MTG were detected as described below. Besides, the cell density and the activity of MTG were measured once every 12 h at different inducted temperatures (i.e. 20, 25 and 30 °C).

The activity of MTG was measured as Yurimoto described [21]. The MTG enzyme activity was defined as follows: catalytic substrate (N-CBZ-Gln-Gly) to generate 1 μmol glutamine hydroxamate (hydroxamic acid) at 37 °C as one enzyme activity unit. Protein concentrations were determined by using a Bradford Protein Assay Kit (TaKaRa) and the standard protein was bovine serum protein (Thermo) [21].

### Expression of engineering strain *pro*/rDNA-*mtg* in flask

The strain GS115 (*pro/*rDNA*-mtg*) of higher enzyme activity was cultured in 100 mL BMGY medium, as described above until the OD600 reached 6–7, and controlled at 28 °C. Then they were harvested and cultured in 200 mL of BMMY medium containing 1% methanol (initial OD600 reached 1.2–1.5) and maintained at 25 °C. 1% methanol (*V*/*V*) was added into BMMY medium once every 24 h for continuous induction for 72 h. The supernatant was collected once every 24 h, centrifuged at 12000 g for 30 mins, and analyzed by SDS-PAGE and Western blot under non-reducing condition. The enzyme activity was detected and verified as described above.

## Additional files

Additional file 1: **Figure S1.**Three-dimensional structure of MTG (a) and pro-MTG (b) from *S. mobaraesis* as determined by PyMOL. The catalytic triad C64-D255-H274 was represented by light blue, red and dark blue, respectively. (TIF 275 kb)

Additional file 2: Sequences of *mtg* and *pro* genes. (DOCX 17 kb)

Additional file 3: **Figures S2, S3.** Secondary structure diagram of mRNA of *mtg* and *pro.* **Figure S2.** The secondary structure diagram of mRNA of *mtg*-WT and *mtg*-Opt. **Figure S3.** The secondary structure diagram of mRNA of *pro*-WT and *pro*-Opt. (ZIP 93 kb)

Additional file 4: **Figure S4.** The product of gene amplification of *pro* and *mtg* in GS115 (*pro*/ rDNA-*mtg*).1,3,5,7: *pro* was amplified by Fw-*pro*(P3) and Rv -*pro* (P4)primers; 2,4,6,8: *mtg* was amplified by Fw-*mtg*(P1) and 3’AOX primers; 9,10: negative controls; M: DNA Marker. (TIF 365 kb)

Additional file 5: **Figure S5.** Standard curves of *gap and mtg gene*. (DOCX 101 kb)

Additional file 6: **Table S1, Figure S3.** Effect on expression and enzyme activity of *mtg* gene copies. **Table S1.** The MTG activity in different copy strains. **Figure S6.** Detection of protein expression in strains with different *mtg* copy by Western blotting. The protein (MTG) in 20 μl of culture supernatant were separated by Western blot analysis (anti-MTG). Lane 1–3: *mtg*-2c, lane 4–6: *mtg*-3c, lane 7–9: *mtg*-6c. (ZIP 48 kb)

## Data Availability

The datasets supporting the conclusions of this article are included with in the article and its additional files. All strain materials were obtained from Anhui Medical College, Hefei, China.

## Abbreviations

*Ct*: Cycle threshold
rDNA: ribosomal DNA
rDNAnc: non-coding rDNA
RFU: relative fluorescence units
