# Supplementary figures and images for: Improved the expression level of active transglutaminase by directional increasing copy of mtg gene in Pichia pastoris
Source: BMC Biotechnol. 2019 Jul 30;19:54. doi: 10.1186/s12896-019-0542-6 (PMC6668168; doi:10.1186/s12896-019-0542-6)

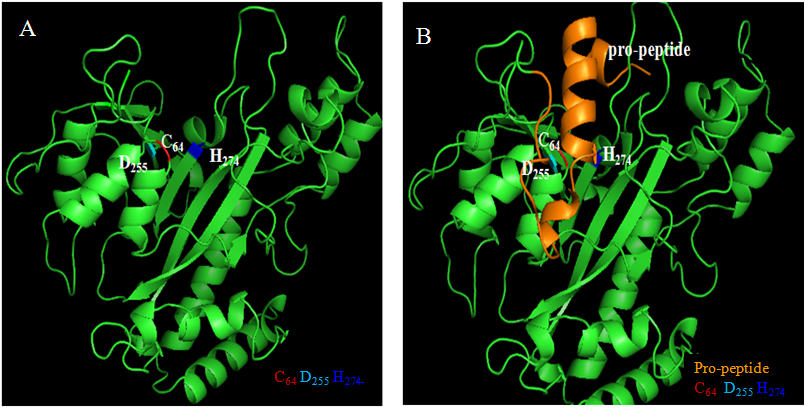

Supplement: Supplementary file 1 — Figure S1.Three-dimensional structure of MTG (a) and pro-MTG (b) from S. mobaraesis as determined by PyMOL. The catalytic triad C64-D255-H274 was represented by light blue, red and dark blue, respectively. (TIF 275 kb) [file 12896_2019_542_MOESM1_ESM.tif]

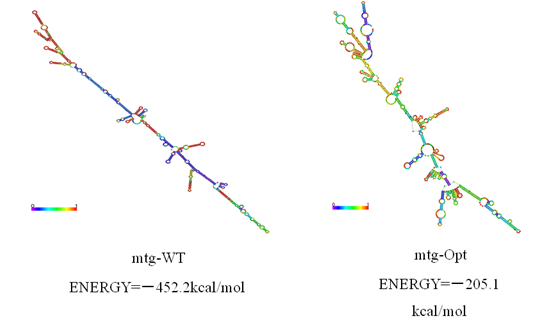

Supplement: Supplementary file 3 — Figures S2, S3. Secondary structure diagram of mRNA of mtg and pro. Figure S2. The secondary structure diagram of mRNA of mtg-WT and mtg-Opt. Figure S3. The secondary structure diagram of mRNA of pro-WT and pro-Opt. (ZIP 93 kb) [file 12896_2019_542_MOESM3_ESM.zip › Additional file 3 Figure S2.tif]

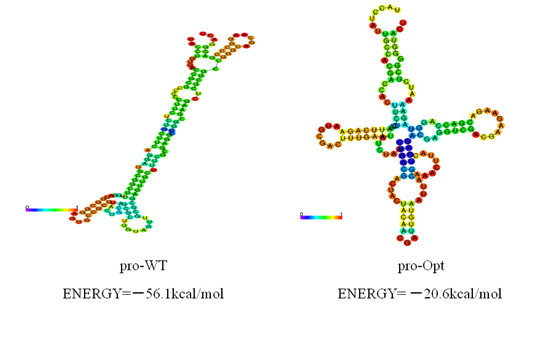

Supplement: Supplementary file 3 — Figures S2, S3. Secondary structure diagram of mRNA of mtg and pro. Figure S2. The secondary structure diagram of mRNA of mtg-WT and mtg-Opt. Figure S3. The secondary structure diagram of mRNA of pro-WT and pro-Opt. (ZIP 93 kb) [file 12896_2019_542_MOESM3_ESM.zip › Additional file 3 Figure S3.tif]

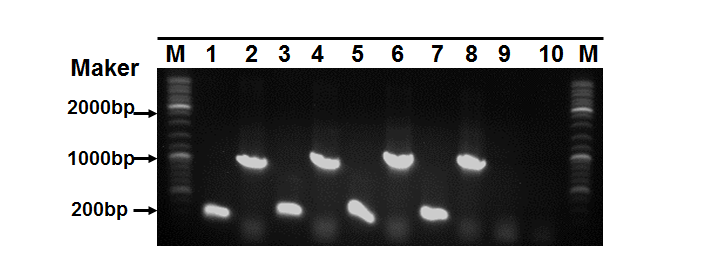

Supplement: Supplementary file 4 — Figure S4. The product of gene amplification of pro and mtg in GS115 (pro/ rDNA-mtg).1,3,5,7: pro was amplified by Fw-pro(P3) and Rv -pro (P4)primers; 2,4,6,8: mtg was amplified by Fw-mtg(P1) and 3’AOX primers; 9,10: negative controls; M: DNA Marker. (TIF 365 kb) [file 12896_2019_542_MOESM4_ESM.tif]

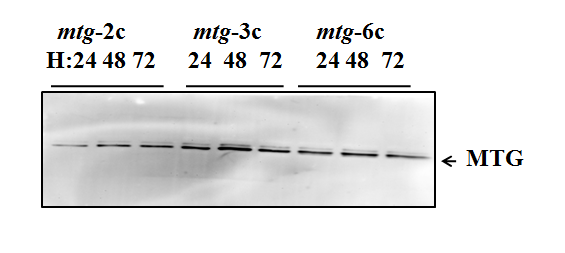

Supplement: Supplementary file 6 — Table S1, Figure S3. Effect on expression and enzyme activity of mtg gene copies. Table S1. The MTG activity in different copy strains. Figure S6. Detection of protein expression in strains with different mtg copy by Western blotting. The protein (MTG) in 20 μl of culture supernatant were separated by Western blot analysis (anti-MTG). Lane 1–3: mtg-2c, lane 4–6: mtg-3c, lane 7–9: mtg-6c. (ZIP 48 kb) [file 12896_2019_542_MOESM6_ESM.zip › Additional file 6 .Figure S6.tif]
