# Supplementary material for: Improved the expression level of active transglutaminase by directional increasing copy of mtg gene in Pichia pastoris
Source: BMC Biotechnol. 2019 Jul 30;19:54. doi: 10.1186/s12896-019-0542-6 (PMC6668168; doi:10.1186/s12896-019-0542-6)
Supplement: Supplementary file 2 — Sequences of mtg and pro genes. (DOCX 17 kb) [file 12896_2019_542_MOESM2_ESM.docx]

**Additional file 2:** **Sequences of *mtg* and *pro* genes**

**一、Sequence Listing**

**1、mtg-Wild (Length:993, GC%:64.48)**

GACTCCGACGACAGGGTCACCCCTCCCGCCGAGCCGCTCGACAGGATGCCCGACCCGTACCGTCCCTCGTACGGCAGGGCCGAGACGGTCGTCAACAACTACATACGCAAGTGGCAGCAGGTCTACAGCCACCGCGACGGCAGGAAGCAGCAGATGACCGAGGAGCAGCGGGAGTGGCTGTCCTACGGCTGCGTCGGTGTCACCTGGGTCAATTCGGGTCAGTACCCGACGAACAGACTGGCCTTCGCGTCCTTCGACGAGGACAGGTTCAAGAACGAGCTGAAGAACGGCAGGCCCCGGTCCGGCGAGACGCGGGCGGAGTTCGAGGGCCGCGTCGCGAAGGAGAGCTTCGACGAGGAGAAGGGCTTCCAGCGGGCGCGTGAGGTGGCGTCCGTCATGAACAGGGCCCTGGAGAACGCCCACGACGAGAGCGCTTACCTCGACAACCTCAAGAAGGAACTGGCGAACGGCAACGACGCCCTGCGCAACGAGGACGCCCGTTCCCCGTTCTACTCGGCGCTGCGGAACACGCCGTCCTTCAAGGAGCGGAACGGAGGCAATCACGACCCGTCCAGGATGAAGGCCGTCATCTACTCGAAGCACTTCTGGAGCGGCCAGGACCGGTCGAGTTCGGCCGACAAGAGGAAGTACGGCGACCCGGACGCCTTCCGCCCCGCCCCGGGCACCGGCCTGGTCGACATGTCGAGGGACAGGAACATTCCGCGCAGCCCCACCAGCCCCGGTGAGGGATTCGTCAATTTCGACTACGGCTGGTTCGGCGCCCAGACGGAAGCGGACGCCGACAAGACCGTCTGGACCCACGGAAATCACTATCACGCGCCCAATGGCAGCCTGGGTGCCATGCATGTCTACGAGAGCAAGTTCCGCAACTGGTCCGAGGGTTACTCGGACTTCGACCGCGGAGCCTATGTGATCACCTTCATCCCCAAGAGCTGGAACACCGCCCCCGACAAGGTAAAGCAGGGCTGGCCG

**2、mtg- Optimized ( Length:993, GC%:39.92 )**

# GATTCTGATGATAGAGTTACTCCACCTGCTGAACCTTTGGATAGAATGCCAGATCCTTACAGACCATCTTATGGTAGAGCTGAGACTGTTGTTAACAACTACATCAGAAAGTGGCAACAAGTTTACTCTCATAGAGATGGTAGAAAACAACAAATGACTGAAGAGCAAAGAGAATGGTTGTCTTACGGTTGTGTTGGTGTTACTTGGGTTAACTCTGGTCAATATCCTACTAATAGATTGGCTTTTGCTTCTTTCGATGAAGATAGATTCAAGAACGAGTTGAAAAATGGTAGACCAAGATCTGGTGAAACTAGAGCTGAATTCGAGGGTAGAGTTGCTAAGGAGTCTTTTGATGAAGAGAAAGGTTTCCAAAGAGCTAGAGAAGTTGCTTCTGTTATGAACAGAGCTTTGGAAAATGCTCACGATGAGTCTGCTTACTTGGATAACTTGAAGAAAGAATTGGCTAACGGTAATGATGCTTTGAGAAACGAGGATGCTAGATCTCCTTTTTATTCTGCTTTGAGAAATACTCCATCTTTCAAGGAGAGAAACGGTGGTAATCATGATCCTTCTAGAATGAAGGCTGTTATCTACTCTAAACACTTTTGGTCTGGTCAAGATAGATCTTCTTCTGCTGATAAGAGAAAATATGGAGATCCAGATGCTTTCAGACCAGCTCCTGGTACTGGTTTGGTTGATATGTCTAGAGATAGAAACATTCCAAGATCTCCTACTTCTCCAGGTGAAGGTTTTGTTAATTTCGATTACGGTTGGTTTGGTGCTCAAACTGAGGCTGATGCTGATAAAACTGTTTGGACTCATGGTAACCATTACCACGCTCCTAATGGTTCTTTGGGTGCTATGCACGTTTATGAATCTAAGTTCAGAAACTGGTCTGAGGGTTACTCTGATTTCGATAGAGGTGCTTACGTTATCACTTTCATTCCTAAGTCTTGGAATACTGCTCCAGATAAGGTTAAACAAGGTTGGCCA

# 3、pro-Wild（Length:135, GC%:64.87)

GACAATGGCGCGGGGGAAGAGACGAAGTCCTACGCCGAAACCTACCGCCTCACGGCGGATGACGTCGCGAACATCAACGCGCTCAACGAAAGCGCTCCGGCCGCTTCGAGCGCCGGCCCGTCGTTCCGGGCCCCC

**4、pro -Optimized （Length:135, GC%:40.00)**

GATAACGGTGCTGGTGAAGAGACTAAGTCTTACGCTGAAACTTATAGATTGACTGCTGATGATGTTGCTAACATTAATGCTTTGAATGAGTCTGCTCCAGCTGCTTCTTCTGCTGGTCCATCTTTTAGAGCCCCA
