# Supplementary material for: Improved the expression level of active transglutaminase by directional increasing copy of mtg gene in Pichia pastoris
Source: BMC Biotechnol. 2019 Jul 30;19:54. doi: 10.1186/s12896-019-0542-6 (PMC6668168; doi:10.1186/s12896-019-0542-6)
Supplement: Supplementary file 5 — Figure S5. Standard curves of gap and mtg gene. (DOCX 101 kb) [file 12896_2019_542_MOESM5_ESM.docx]

**Additional file 5: Figure. S5 Standard curves of *gap and mtg gene***

**1*、***standard curve of ***gap*** gene

Fig. S5-1 amplification curve and standard curve ***gap*** gene

**2、**standard curve of ***mtg*** gene

Fig. S5-2 amplification curve and standard curve ***mtg*** gene
