# Supplementary material for: Improved the expression level of active transglutaminase by directional increasing copy of mtg gene in Pichia pastoris
Source: BMC Biotechnol. 2019 Jul 30;19:54. doi: 10.1186/s12896-019-0542-6 (PMC6668168; doi:10.1186/s12896-019-0542-6)
Supplement: Supplementary file 6 — Table S1, Figure S3. Effect on expression and enzyme activity of mtg gene copies. Table S1. The MTG activity in different copy strains. Figure S6. Detection of protein expression in strains with different mtg copy by Western blotting. The protein (MTG) in 20 μl of culture supernatant were separated by Western blot analysis (anti-MTG). Lane 1–3: mtg-2c, lane 4–6: mtg-3c, lane 7–9: mtg-6c. (ZIP 48 kb) [file 12896_2019_542_MOESM6_ESM.zip › Additional file 6. TableS1.docx]

**Table S1 The MTG activity in different copy strains**

| Strain  (*pro*/rDNA*-mtg*) | Total activity  （U/mL） | Protein conc (mg/L) | Specific activity  （U/mg） |
| --- | --- | --- | --- |
| mtg-2c mtg-3c  mtg-6c | 0.37±0.02 ^b^  1.41 ±0.08 ^a^  0.28±0.03 ^b^ | 119.7±3.24^b^  173.3±5.85 ^a^ 101.3±3.21^b^ | 3.11±0.71^b^  8.15±0.61 ^a^  2.77±0.47^b^ |
|  |  |  |  |

Datas are presented as mean ± SD of triplicate observations. Values with different superscripts are significantly different (*p* < 0.05)
